# Association between premenstrual syndrome and eating disturbance in college students: a cross-sectional study

**DOI:** 10.1186/s12905-024-03158-0

**Published:** 2024-06-07

**Authors:** Yuka Yoshinari, Saori Morino, Yuki Shinohara, Chang Yu Chen, Miyu Onishi, Yuna Akase, Momoko Nagai-Tanima, Tomoki Aoyama

**Affiliations:** 1 Department of Physical Therapy, Human Health Sciences, Graduate School of Medicine, Kyoto University, 53 Kawahara-cho, Shogoin, Sakyo-ku, Kyoto, 606-8507 Japan; 2 Department of Rehabilitation Science, School of Medicine, Osaka Metropolitan University, 3-7-30 Habikino, Habikino-shi, Osaka 583-8555 Japan

**Keywords:** Premenstrual syndrome, Eating behavior, Eating disturbance, College students

## Abstract

**Background:**

Premenstrual syndrome (PMS) is a severe problem in women, and a well-balanced diet helps improve PMS symptoms. Eating disturbances are a major health problem in young women. Limited research has explored the correlation between eating behaviors and PMS symptoms in Japan. This study aimed to compare eating disturbances and the severity of PMS symptoms in college students.

**Methods:**

This study was conducted among female college students using an online questionnaire. The questionnaire included basic information (age, height, and weight), PMS symptoms, and eating behaviors assessed using the Eating Attitudes Test 26.

**Results:**

The proportion of those with PMS symptoms who were disturbed by PMS symptoms was significantly higher in the group with eating disturbance. Those who were affected by the physical symptoms of PMS had significantly higher scores on the subscales related to diet, bulimia and food preoccupation.

**Conclusion:**

The results showed an association between PMS symptom severity and eating disturbance. The findings of this study indicate that individuals with eating disturbances may experience adverse effects on PMS symptoms, even in cases where weight is not at the extremes of excessive underweight or obesity.

## Background

Premenstrual syndrome (PMS) is a cyclical pattern of physical and psychological symptoms that appear in the luteal phase after ovulation and disappear soon after the onset of menstruation symptoms [1, 2]. The frequently reported symptoms include irritability, fatigue, anxiety or tension, malaise, changes in appetite and sleep, breast tenderness, headache, and poor concentration [3].

Various studies have been conducted on the relationship between PMS symptoms and lifestyle habits, with reports stating that the prevalence of PMS is higher in those who do not have a regular exercise routine [4] and that missing breakfast, coffee, and alcohol consumption are associated with worsening PMS symptoms in female students aged 17 years and older [5]. Dietary content has also been reported to be associated with PMS, and a well-balanced diet is said to help improve PMS symptoms and is often used as the first step in PMS treatment [6]. There are also reports that vitamin D [7] and zinc [8] intake are associated with decreased PMS risk, and that high fat foods [9] and fast food [10] intake are associated with increased PMS risk, suggesting that diet and PMS symptoms are closely related.

In young women, 80.5% of college students have PMS [11], while in Japan, a study of college and graduate students reported that 90.2% had PMS symptoms [12], suggesting the need for appropriate support and information provision. In addition, 60.4% of female college students who were aware of PMS symptoms reported that PMS interfered with their normal lives, and 35.4% missed school or work because of PMS [13]. Moreover, college students with mild PMS reported lower scores in all domains of quality of life (QOL) than non-PMS students [14], and the impact of PMS symptoms on the lives and QOL of young women is considered problematic. The relationship between PMS and nutrition has been reported [7–9] and is of interest to female students suffering from PMS. Various studies have also been conducted on PMS and diet, including breakfast intake and number of chews [15, 16]. Similar to lifestyle issues related to food, problems with eating behavior are also becoming a growing issue among female students in Japan [17]. In particular, the impact of body image on health is concerning and needs to be considered not only in Japan but worldwide [18].

Based on these findings, we hypothesized that, PMS symptoms may be more severe among young Japanese women with eating disturbance than in those with normal eating behavior, owing to the effects of nutrient imbalance. Although studies have focused on PMS and diet in the context of lifestyle in some countries [15, 16], only a few have reported on eating behaviors [19, 20] and, compared eating behaviors and PMS symptoms in Japan. Therefore, this study aimed to compare eating disorder tendencies and the severity of PMS symptoms among college students.

## Methods

### Study design

This was a cross-sectional survey conducted via online questionnaire. Participants were recruited from Japanese universities from November 2022 to September 2023. The included individuals were female undergraduate and graduate students under the age of 25. The exclusion criterion was psychiatric disorders that were not associated with PMS.

### Participants

This study was conducted among female undergraduate and graduate students using an online questionnaire. The study was fully explained online, and informed consent was obtained by submitting the questionnaire.

The questionnaire was created using a web-based survey service, Survey Monkey (Sun Mateo, California, USA), and cooperation was requested through social networking services. The questionnaire included basic information (age, height, and weight), PMS symptoms, and eating behaviors. This study was approved by the Ethics Committee of the Graduate School of Medicine, Faculty of Medicine, Kyoto University, and its affiliated hospitals (approval number: R2634-2).

The sample size of this study was 171 persons. In the no correspondence t-test, a standard effect size of f = 0.5 was set, and when the probability of alpha error was 5% and that of beta error was 20%, the required sample size was 128 respondents. Assuming an attrition rate of approximately 25% for online questionnaires, based on a previous study [21], the required sample size was 128 ÷ (1-0.25) = 171. Based on this, the planned number of participants was set at 171.

### Questionnaire

#### PMS

PMS symptoms were defined by referring to previous studies [19] and adding the 10 symptoms used in the diagnostic criteria of the American College of Obstetrics and Gynecology [22] to the 8 symptoms reported in previous studies [23–25], totaling 18 symptoms according to expert opinion (Table 1). For each of the 18 symptoms, respondents were asked to select one of three options: “no symptoms,” “symptoms not enough to be bothersome but bothersome,” or “bothersome.” Those who responded that they were “bothersome” by any of the 18 overall PMS symptoms were classified as “disturbed” and the rest were classified as “not disturbed. Similarly, the two groups were divided into two groups according to whether or not they were disturbed by any of the 10 physical symptoms and whether or not they were disturbed by any of the 8 mental symptoms [21–24]. 

**Table 1:** Questionnaire of PMS

Physical symptoms	Psychological symptoms
Breast tenderness or pain	Mood swings
Headaches	Irritability
Lower abdominal distension	Confusion of feelings
Lower abdominal pain	Explosions of anger
Swelling of hands or feet	Anxiety
Increased appetite and overeating	Emergence of desire to be alone
Tiredness and lethargy	Loss of concentration
Back pain	Loss of motivation
Drowsiness	
Acne	

#### Eating behavior

Using a reduced version of the Eating Attitudes Test (EAT) [26], eating behavior was assessed (EAT26 [27]). Garner et al. conducted a factor analysis of the EAT to identify associations between clinical and personality characteristics and EAT questions and identified three factors, and an EAT26 was created by excluding questions that did not fit these factors [27]. The EAT26 has been shown to be valid and reliable for assessing Anorexia Nervosa (AN) [27]. Each question has six levels of selection, with a total score of 3, 2, and 1, and the rest being 0, in order from the most extreme responses with the strongest eating disturbance [26]. The cutoff value of 20 points [27] was used to classify the two groups: those who scored 20 points or more were classified as “having eating disturbance,” and those who scored less than 20 points were classified as “not having eating disturbance.” Based on the factor analysis by Garner et al. [27], the scores for Factor I (dieting), Factor II (bulimia and food preoccupation), and Factor III (oral control) were calculated.

The subscale contents are presented in Table 2. According to previous studies, although there was no significant difference in the total EAT26 scores between individuals with AN who had complications of bulimia and individuals with restricted AN who did not, Factor II scores were significantly higher, and Factor III scores were significantly lower in individuals with AN who had complications of bulimia than in individuals with restricted AN [27].

**Table 2:** EAT26 subscales and their contents

Factor I	Dieting	Avoidance of fatty food Obsession with thinness
Factor II	Bulimia and food preoccupation	Bulimia Thoughts on food
Factor III	Oral control	Self-control on eating Pressure from others to gain weight

Garner et al. [26]

## Analysis

First, the Shapiro–Wilk test was conducted to confirm the normality of the results, followed by the Welch’s t-test to determine if there were differences in basic information (age, height, weight, and BMI) depending on the presence or absence of eating disturbances.

Next, the chi-square test was conducted to examine the relationship between eating disturbance and PMS symptoms, and the proportions of those who were disturbed by PMS overall, PMS physical symptoms, and PMS psychological symptoms were compared between the two groups with and without eating disturbance.

Finally, the Welch’s t-test was conducted to compare the PMS and EAT26 subscale scores. The scores of the three subscales of the EAT26 were compared between the two groups whether they were disturbed by PMS, two groups whether disturbed by PMS physical symptoms, and two groups whether disturbed by PMS psychiatric symptoms.

JMP Pro17 (SAS Institute Inc., Cary, NC, USA) was used for statistical analysis, with a significance level of *p* < 0.05.

## Results

### Basic information

There were 171 online questionnaire respondents, of whom 133 (77.8%) completed the questionnaire. After excluding those with missing responses, 130 (76.0%) responses were included in the data used for this analysis.

Of the 130 participants analyzed, 8 (6.15%) had eating disturbances. There were no significant differences in basic information between the groups with and without eating disturbances (Table 3).

**Table 3:** Eating disturbance in two groups and basic information

	Eating disturbance(*n* = 8)	No eating disturbance (*n* = 122)	*P* value
Age(years)	19.6 ± 0.7	20.1 ± 1.6	0.397
Hight(cm)	158.0 ± 4.9	159.0 ± 5.5	0.618
Weight(kg)	52.3 ± 4.2	51.7 ± 5.6	0.805
BMI (kg/m^2^)	21.0 ± 2.0	20.5 ± 1.9	0.475

Results are presented as Mean ± SD

### Eating disturbance and PMS symptoms

The proportion of those who were disturbed by PMS symptoms was significantly higher in the group with eating disturbances than in the group without eating disturbances.

Similarly, the proportion of those who were disturbed by physical symptoms of PMS, and those who were disturbed by psychological symptoms of PMS were significantly higher in the group with eating disturbances (Table 4).

**Table 4:** Comparison of PMS disturbance proportions in two groups with eating disturbance tendencies

	Eating disturbance(*n* = 8)	No eating disturbance(*n* = 122)	*P* value
Disturbed by PMS	100%(*n* = 8)	59.8%(*n* = 73)	0.023*
Disturbed by physical symptoms of PMS	100%(*n* = 8)	55.7%(*n* = 68)	0.014*
Disturbed by psychological symptoms of PMS	62.5%(*n* = 5)	27.1%(*n* = 33)	0.033*

*p < 0.05

### EAT26 subscale and PMS symptoms

Scores on each subscale of the EAT26 were compared based on the presence or absence of PMS-related disturbances. Of the 18 overall PMS symptoms, 81 (62.3%) respondents reported being disturbed by one or more of the symptoms. Those with disturbances of PMS symptoms had significantly higher Factor II scores than those without disturbances, while Factor I and Factor III scores were not significantly different when comparing those with and without PMS symptom disturbances (Table 5).

The EAT26 subscale scores were also compared according to the presence or absence of disturbances by physical and psychological symptoms of PMS. Factor I and Factor II scores were significantly higher for those who were disturbed by any of the physical symptoms of PMS than for those who were not (Table 6). In contrast, there was no significant difference in the scores of each subscale between the two groups according to the presence or absence of PMS psychiatric symptoms (Table 7).

**Table 5:** Comparison of EAT26 subscale scores according to the presence or absence of PMS disturbance

	Disturbed (*n* = 81)	No disturbance (*n* = 49)	*P* value
FactorI	6.7 ± 5.2	5.3 ± 2.7	0.051
FactorII	1.5 ± 2.6	0.6 ± 1.3	0.012*
FactorIII	1.7 ± 1.8	1.7 ± 1.8	0.876

FactorI Dieting, FactorII: Bulimia and food preoccupation, FactorIII: Oral control. *p < 0.05. Results are presented as Mean ± SD

**Table 6:** Comparison of EAT26 subscale scores according to the presence or absence of physical symptom disturbances

	Disturbed (*n* = 76)	No disturbed (*n* = 54)	*P* value
FactorI	6.8 ± 5.4	5.2 ± 2.6	0.029*
FactorII	1.6 ± 2.7	0.6 ± 1.2	0.005*
FactorIII	1.6 ± 1.6	1.8 ± 2.0	0.559

FactorI Dieting, FactorII: Bulimia and food preoccupation, FactorIII: Oral control. *p < 0.05. Results are presented as Mean ± SD

**Table 7:** Comparison of EAT26 subscale scores according to the presence or absence of psychological symptom disturbance

	Disturbed (*n* = 38)	No disturbed (*n* = 92)	*P* value
FactorI	7.1 ± 5.5	5.8 ± 4.0	0.171
FactorII	1.8 ± 3.1	0.9 ± 1.7	0.112
FactorIII	1.9 ± 2.0	1.6 ± 1.7	0.364

FactorI Dieting, FactorII: Bulimia and food preoccupation, FactorIII: Oral control. Results are presented as Mean ± SD

## Discussion

### PMS and eating disturbance

The results of this study showed that the proportion of those who were disturbed by PMS symptoms, PMS physical symptoms, and PMS mental symptoms were significantly higher in the group with eating disturbances than in the group without eating disturbances.

Regarding nutrients, there are reports that mental and physical symptoms of PMS decrease in the group that consumes zinc gluconate [8], vitamin B6 may be effective in reducing PMS symptoms [28], and vitamin D intake is associated with a lower risk of PMS [7].

In addition, a study examining the relationship between eating disturbances and nutrient intake using the EAT26 reported that the group with eating disturbances had lower intakes of zinc, vitamin B6, and vitamin B12, as well as the three macronutrients compared with that observed in the group without eating disturbance [29].

This may have resulted in a nutrient imbalance in the group with eating disturbances, which may have been associated with worsening PMS symptoms.

One study reported that PMS severity was higher in students who were thin (BMI < 18.5 km/m^2^) than in the other students [5]. Conversely, individuals with obesity (BMI ≥ 25 km/m2) have a higher prevalence of PMS than those with a normal BMI [30]. In the present study, BMI was not correlated with either PMS symptoms (data not shown) or eating disturbance (Table 3). This indicates that other factors involved besides BMI and nutrient bias are involved. After all, for female college students, the influence of body image through social media [18] has an impact on PMS symptoms as well as issues such as being thin. It is particularly significant that among PMS symptoms, physical changes had a greater impact than mental changes. Given these differences between previous reports and the findings of the present study, eating behaviors other than conventional nutrition and BMI may have influenced PMS, and a different perspective for health guidance for PMS may be needed.

### PMS physical symptoms and bulimia tendency

The results of this study suggest that scores on Factor II of the EAT26 or overeating are related to PMS symptoms, particularly physical symptoms.

Previous studies reported that bread, pasta, sweet, high-fat meat, and salty snacks are the foods most commonly consumed by non-purge binge [31]. The Yale Food Addiction Scale, which was created to assess food dependence, particularly the intake of high-fat and high-sugar foods, has been reported to correlate with EAT26 scores and may also be a predictor of overeating [32]. These findings suggest that those who score higher on Factor II of the EAT26 may consume more high-sugar and high-fat foods than those who score lower on Factor II of the EAT26.

Additionally, it has been reported that the intake of foods high in calories, fat, sugar, and salt is associated with an increased risk of PMS symptoms [9] and that there is a positive correlation between the amount of fast food, instant noodles, chocolate, and candy consumed and PMS severity [10].

Based on these findings, a high intake of high-fat and high-sugar foods may have contributed to the worsening of PMS symptoms among those who scored high on Factor II of EAT26. In addition, since the PMS physical symptoms in this study included increased appetite and overeating, it is possible that Factor I and II scores, including overeating and thoughts about food, were particularly associated with the worsening of PMS physical symptoms.

On the other hand, there was no association between the scores of Factors I and III of the EAT26 and PMS symptoms; Factor I is said to be unrelated to overeating, while Factor III is said to be negatively correlated with overeating [27], and the present results of Factors I and III were not related to the intake of high-fat and high-sugar foods, which have been suggested to affect PMS.

Limitations of the Study and Future Prospects.

First, because of the cross-sectional study design, we can mention an association between PMS and eating behavior, not a causal relationship. Second, the small sample size poses a major limitation for generalization. In particular, the survey of only college students makes comparisons with other generations difficult. Third, the self-reported questionnaire used in this study may cause recall bias. Furthermore, eating behavior may have changed over time, making it challenging to accurately capture the eating behavior of modern female college students. Forth, symptoms other than PMS could not be clearly distinguished. Previous studies have demonstrated that the sleep, nutrition and work stress have been identified as major PMS aggravating factors in other population [24]. However, high school and college students compared with working adults are more concerned about body image and social media opinions, and their eating behavior may be significantly influenced. In the future, comparisons of eating behavior and other PMS items in a larger population may contribute to the improvement and prevention of PMS.

## Conclusions

The results of this study indicate that eating behavior has an impact on PMS symptoms in female college students, even if it does not affect actual body composition such as BMI. Future comprehensive comparisons of eating behavior, body image, body composition and nutritional status with PMS symptoms will further clarify these relationships.

## Data Availability

The datasets used and/or analyzed during the current study are available from the corresponding author on reasonable request.

## Abbreviations

PMS: Premenstrual syndrome
QOL: Quality of Life
AN: Anorexia Nervosa
EAT: Eating Attitudes Test
